# Touch hunger: trajectory and predictors of longing for physical contact during the COVID-19 pandemic in people with and without psychiatric disorders

**DOI:** 10.1192/j.eurpsy.2024.764

**Published:** 2024-08-27

**Authors:** S. E. Mann, A. L. Kok, N. Rius Ottenheim, E. J. Giltay, B. W. Penninx

**Affiliations:** ^1^Psychiatry, Leiden University Medical Center, Leiden; ^2^Psychiatry, Amsterdam University Medical Center, Amsterdam; ^3^Health Campus, Leiden University, Den Haag, Netherlands

## Abstract

**Introduction:**

Little is known about touch hunger (longing for physical contact) during the COVID-19 pandemic, particularly for people with pre-existing mental health disorders.

**Objectives:**

We aim to investigate the dynamics of touch hunger in people with and without depressive, anxiety, or obsessive-compulsive disorders during the COVID-19 pandemic, and the potential predictors for touch hunger during lockdown.

**Methods:**

Data were aggregated from three Dutch ongoing prospective cohorts with similar methodology for data collection. We included participants with pre-pandemic data gathered during 2006–2016, and who completed up to 9 online questionnaires between October 2020 and February 2022. We compared trajectories between subgroups with different pre-pandemic chronicity of disorders and healthy controls using linear mixed models. Sociodemographic, clinical (number and type of mental health disorders, personality traits) and COVID-19-related variables were analysed as predictors of touch hunger using multivariate linear regression analyses.

**Results:**

We included 1061 participants with (*n* = 811) and without (*n* = 250) mental health disorders. In all groups, touch hunger increased during lockdown (Fig. 1). Extraversion (β = 0.256, P <0.001), social distancing due to COVID-19 anxiety (β = 0.122, P = 0.001) and death of a close contact from COVID-19 (β = 0.073, P = 0.02) predicted higher touch hunger, while living with a partner (β = -0.109, P = 0.004) or with a partner and children (β = -0.147, P <0.001) were protective factors for touch hunger. Remarkably, pre-pandemic mental disorders did not predict touch hunger during lockdown.

**Image:**

**Figure:**
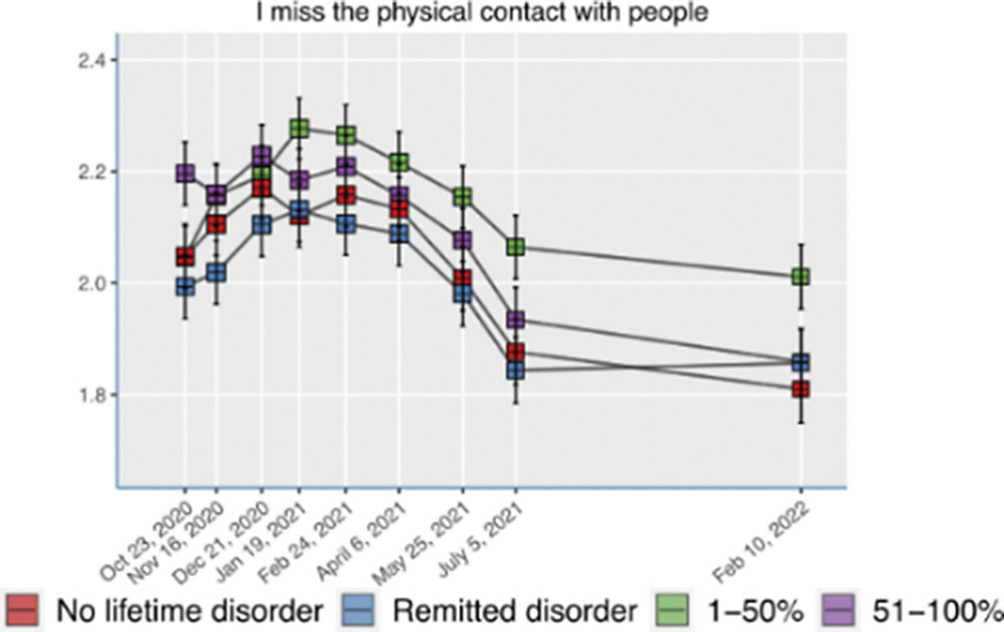

**Conclusions:**

Social distancing measures have important psychological and emotional implications, as our study showed an increase in touch hunger during lockdown, which did not differ between people with and without mental health disorders. Extroverted individuals may benefit most from interventions aimed at addressing their need for physical contact during times of crisis.

**Disclosure of Interest:**

None Declared

